# Magnetic resonance imaging findings in autoimmune hepatitis: how frequent and reproducible are they?

**DOI:** 10.1590/0100-3984.2023.0044

**Published:** 2023

**Authors:** Natália Borges Nunes Gomes, Ulysses S. Torres, Gabriella Souza e Silva, Perla Oliveira Schulz Mamone, Maria Lucia Cardoso Gomes Ferraz, Giuseppe D’ippolito

**Affiliations:** 1 Escola Paulista de Medicina da Universidade Federal de São Paulo (EPM-Unifesp), São Paulo, SP, Brazil.; 2 Grupo Fleury, São Paulo, SP, Brazil.

**Keywords:** Abdomen, Hepatitis, autoimmune, Liver, Magnetic resonance imaging, Diagnostic techniques and procedures, Abdome, Hepatite autoimune, Fígado, Ressonância magnética, Técnicas e procedimentos diagnósticos

## Abstract

**Objective:**

To determine the frequency and interobserver reproducibility of the magnetic resonance imaging (MRI) features considered diagnostic for autoimmune hepatitis.

**Materials and Methods:**

Two abdominal radiologists, blinded to pathology data, reviewed the MRI examinations of 20 patients with autoimmune hepatitis, looking for liver enhancement, lymphadenopathy, portal hypertension, and chronic liver disease. The pattern of liver fibrosis was categorized as reticular, confluent, or mixed. Interobserver agreement was assessed by calculating intraclass correlation coefficients and kappa statistics.

**Results:**

The most common abnormal finding on MRI was surface nodularity (in 85%), followed by liver fibrosis with a reticular pattern (in 80%)—categorized as mild (in 25.0%), moderate (in 43.8%), or severe (in 31.2%)—; heterogeneous liver enhancement (in 65%); splenomegaly (in 60%); caudate lobe enlargement (in 50%); and lymphadenopathy (in 40%). The interobserver agreement was almost perfect for surface nodularity (0.83), ascites (0.89), and liver volume (0.95), whereas it was just slight and fair for the degree of fibrosis and for heterogeneous liver enhancement (0.12 and 0.25, respectively). It was also slight and fair for expanded gallbladder fossa and enlarged preportal space (0.14 and 0.36, respectively), both of which are indicative of chronic liver disease.

**Conclusion:**

The interobserver agreement was satisfactory for surface nodularity (the most prevalent abnormal MRI finding), ascites, liver volume, and splenomegaly. Conversely, it was only slight or fair for common but less objective criteria.

## Abstract (pt)

**Objetivo:**

Determinar a frequência e reprodutibilidade interobservador das características de imagem por ressonância magnética na hepatite autoimune.

**Materiais e Métodos:**

Dois radiologistas abdominais, cegos para dados patológicos, revisaram ressonâncias magnéticas de 20 pacientes com hepatite autoimune quanto ao realce hepático, linfadenopatia, hipertensão portal e doença hepática crônica. A fibrose foi classificada como reticular, confluente ou ambas. A concordância interobservador foi avaliada por coeficientes de correlação intraclasse e estatística kappa.

**Resultados:**

O achado anormal mais comum foi nodularidade superficial (85%), seguido de fibrose reticular hepática (80%) — leve (25%), moderada (43,8%), grave (31,2%) —, realce heterogêneo (65%), esplenomegalia (60%), aumento do lobo caudado (50%) e linfadenopatia (40%). A concordância interobservador foi quase perfeita para nodularidade superficial (0,83), ascite (0,89) e volume hepático (0,95); entretanto, foi apenas leve (0,12) e razoável (0,25) para grau de fibrose e realce heterogêneo, respectivamente. Também foi leve (0,14) ou regular (0,36) para achados de doença hepática crônica, como fossa da vesícula biliar expandida e espaço pré-portal alargado, respectivamente.

**Conclusão:**

A concordância geral foi satisfatória para nodularidade superficial (achado anormal mais prevalente), ascite, volume hepático e esplenomegalia. Critérios frequentes, porém menos objetivos, tiveram apenas concordância leve a razoável.

## INTRODUCTION

Autoimmune hepatitis (AIH) is a rare disease related to chronic inflammation of the liver, and the prognosis is poor in the absence of treatment^(1^. The treatment has the main goal of achieving clinical, biochemical, and histologic remission. Even with clinical improvement, histological remission of inflammation would be necessary in order to justify the discontinuation or reduction in the dosage of the drugs employed^(2^.

Making a diagnosis of AIH is challenging because there is no one pathognomonic feature or laboratory marker sensitive and specific enough to define it. Therefore, the best and most widely used method for diagnosing AIH nowadays is the International Autoimmune Hepatitis Group simplified score^(3^, which is based on four independent variables: histology, autoantibodies, immunoglobulin G levels, and exclusion of markers of viral infection.

Historically, imaging examinations have not contributed to the diagnosis of AIH, because the findings may be variable and nonspecific, most of them being related to chronic liver disease (CLD), as previously described^(4^. Because biopsy is still the best method for accurately demonstrating liver tissue inflammation, imaging studies have played a limited role in the clinical management of AIH, although the concept of “virtual biopsy” is emerging rapidly with new, advanced imaging methods^(4^ and has already shown promising results in the assessment of liver fibrosis in AIH using noninvasive imaging methods. Therefore, the relevance of conventional imaging lies mainly in excluding overlapping syndromes—e.g., bile duct injury, including destructive cholangitis, in conjunction with otherwise classical features of AIH, may constitute an overlap syndrome between AIH and primary biliary cholangitis; and bile duct injury, manifested by ductopenia, portal fibrosis, and portal edema, suggests an overlap syndrome with primary sclerosing cholangitis, etc.^(5^—and in assessing cirrhosis complications, such as screening for hepatocellular carcinoma^(2,4^.

Although several previous studies have assessed specific morphological changes seen on imaging in liver disease and have attempted to identify correlations with different etiologies^(6,7^, only a few studies have assessed the magnetic resonance imaging (MRI) features of AIH^(8,9^. However, the latter studies have been limited, one by not having histopathologic confirmation in all patients, as well as possibly including some overlap syndromes in their sample^(8^, and the other by evaluating a small sample comprising only 12 MRI examinations^(9^. Other studies related to imaging in AIH were directed toward the computed tomography (CT) assessment of very specific characteristics, such as hypervascular nodules^(10^, the evaluation of CT imaging features for the diagnosis of autoimmune acute liver failure^(11^, or the characterization of overlap syndromes on MRI in autoimmune liver diseases^(12^. Considering all the data available in the current literature regarding the associations between morphological changes and the etiology of liver disease, a subjective evaluation of the liver through imaging with a focus on the etiology is paramount in the clinical practice of radiologists, and better comprehension of the nature and prevalence of such changes in AIH would therefore be of interest. To our knowledge, there have been no studies systematically reporting the reproducibility of the full spectrum of findings.

Considering the greater availability of conventional MRI and its lower cost in comparison with the use of advanced MRI sequences, as well as the scarcity of studies on this subject, we aimed to determine the frequency and interobserver reproducibility of the spectrum of diagnostic MRI features in a sample of patients with a confirmed diagnosis of AIH.

## MATERIALS AND METHODS

The study was approved by the Research Ethics Committee of the Escola Paulista de Medicina da Universidade Federal de São Paulo (Reference No. 1066/2018), in the city of São Paulo, Brazil. Because of the retrospective, descriptive, non-interventional nature of the study, the requirement for informed consent was waived. We reviewed the hepatology outpatient registry database at our institution to identify all patients with AIH who had undergone MRI of the liver between January 2009 and December 2019; that corresponds to the period in which the epidemiological and imaging databanks compiled by the multidisciplinary team of hepatologists and radiologists in our institution were most systematically and consistently organized, without disruptions or interruptions, which allowed the proper selection of consecutive patients.

A total of 26 patients were identified. Six patients with concomitant diseases—hemochromatosis, primary biliary cholangitis, or primary sclerosing cholangitis—were excluded. Therefore, the final sample comprised 20 patients, of whom 16 were women. The mean age was 45 years (range 16–76 years). All of the patients included had a diagnosis of AIH based on a combination of clinical, biochemical, immunological, and histopathological parameters, in accordance with the diagnostic criteria defined by the International Autoimmune Hepatitis Group^(3,13^.

### MRI technique

All MRI studies were executed in a 1.5-T scanner (Gyroscan Intera; Philips Medical Systems, Best, The Netherlands) or in a 3.0-T scanner (Skyra 3T; Siemens Medical Systems, Erlangen, Germany), and a standard liver protocol was followed. The protocol included the following: axial unenhanced T1-weighted sequence; axial in-phase and out-of-phase gradient-echo sequences; axial T2-weighted single-shot turbo spin-echo sequence; axial fat-suppressed T2-weighted sequence, and axial diffusion-weighted imaging sequences (b-values: 0, 50, 400, and 800 s/mm^2^). Axial contrast-enhanced images were acquired after injection of 0.1 mmol/kg of the extracellular contrast agent gadoterate meglumine (Dotarem; Guerbet, Villepinte, France) into a peripheral vein at an infusion rate of 2 mL/s. Contrast-enhanced images of the liver were obtained in the axial plane, in the arterial phase (25–35 s), portal venous phase (65–70 s), and delayed phase (3–5 min).

### Image analysis

#### Determination of the frequency of diagnostic MRI features

Two radiologists (with 1 and 3 years of experience in abdominal imaging, respectively), who were blinded to the pathology and clinical data, retrospectively reviewed all MRI examinations independently. In cases in which there was disagreement regarding the frequency of diagnostic MRI features, a senior radiologist (with 30 years of experience in abdominal imaging) resolved the issue.

To better define the morphology of the liver, the following findings were evaluated subjectively: surface nodularity, expanded gallbladder fossa, and enlarged preportal space. Findings related to portal hypertension were also evaluated^(8^: dilatation of the portal vein (> 12 mm in coronal axis) and splenic vein (> 9 mm in axial axis); portal and splenic vein thrombosis; collateral vessels; splenomegaly; and ascites. Caudate lobe enlargement was defined on the basis of the modified caudate-right lobe ratio proposed by Awaya et al.^(14^, with a cutoff value of greater than 0.90 to indicate hypertrophy. To identify splenomegaly, the splenic index, calculated as the product of the longitudinal, transverse, and anteroposterior axes of the spleen (abnormal > 480), was employed^(15^.

Liver fibrosis was categorized as reticular, confluent, or mixed, being characterized exclusively on the basis of the imaging characteristics, without histopathological correlation, as previously proposed in the literature^(8^. When the fibrosis had a reticular pattern, it was subcategorized as mild, moderate, or severe. The reticular pattern was defined as fine lines with low signal intensity on out-of-phase MRI sequences, showing pronounced contrast enhancement in the delayed phase. As in previous studies, a four-point scoring system was used in order to evaluate the extent of such fibrosis, as follows: 0, none; 1, mild (defined as a thin network of linear fibrous tissue with a diameter < 2 mm, without obvious surface nodularity); 2, moderate (defined as linear fibrotic bands measuring 2–5 mm, with surface nodularity caused by intervening bands of fibrosis); and 3, severe (defined as thick fibrotic bands measuring > 5 mm). The confluent pattern of fibrosis was defined as a region of amorphous fibrosis tissue > 2 cm in diameter that showed the same characteristics as the reticular pattern on unenhanced and contrast-enhanced MRI sequences. When the reticular and confluent patterns were both present, the fibrosis was categorized as mixed^(8^.

On the basis of contrast-enhanced MRI sequences acquired in the arterial phase, liver enhancement was categorized as homogeneous (regular) or heterogeneous (patchy). As defined by Semelka et al.^(16^, a homogeneous pattern of liver enhancement is characterized by uniform parenchymal enhancement, whereas a patchy pattern of liver enhancement pattern is characterized by heterogeneous or cloud-like parenchymal enhancement. Liver volume was calculated as the product of the maximum diameters of the liver, divided by the constant 3.63^(17^. Hepatic steatosis was diagnosed by observing the relative in-phase and out-of-phase values for the liver and spleen: if the liver signal intensity loss was > 10%, the diagnosis was made^(18^. Liver nodules were detected and characterized on the basis of previously reported criteria, with a special focus on regenerative nodules that are hypervascular and on hepatocellular carcinoma^(19^.

The intrahepatic bile duct was categorized as dilated when the diameter was greater than 3 mm, as determined from the T2-weighted or delayed-phase contrast-enhanced MRI sequences^(8^. Intrahepatic biliary dilatation was categorized as general or segmental, depending on whether it was diffuse throughout the liver parenchyma or involved only one of its segments or subsegments, respectively^(10^. Periportal and portacaval lymphadenopathy (short axis > 1 cm) were also assessed.

#### Determination of interobserver agreement

All of the MRI features mentioned above, as assessed by readers 1 and 2, were also analyzed in terms of interobserver agreement, as further explained below.

### Statistical analysis

For the calculation of the mean and standard deviation for each quantitative variable, the mean of the values assigned by readers 1 and 2 was considered. Cohen’s kappa **(κ)** or the intraclass correlation coefficient (ICC) was used in order to analyze reproducibility between the readers, depending on the type of variable analyzed. The choice of tests was based on the guidelines established by Kottner et al.^(20^.

The weighted and unweighted Cohen’s κ values were used for ordinal and nominal variables, respectively. The ICC was applied to assess the reproducibility of numerical variables. The choice of the ICC type was based on the guidelines established by Koo et al.^(21^. Given the limitations of the κ statistic for homogeneous samples, the Gwet AC1 statistic was also calculated for categorical variables^(22^.

The classification of the κ and AC1 statistics was based on Landis et al.^(23^: 0.00–0.20 = slight agreement; 0.21–0.40 = fair agreement; 0.41–0.60 = moderate agreement; 0.61–0.80 = substantial agreement; and 0.81–1.00 = almost perfect agreement. The classification of the ICC values was based on Koo et al.^(21^: < 0.50 = poor agreement; 0.50–0.75 = moderate agreement; 0.75–0.90 = good agreement; and > 0.90 = excellent agreement.

In all analyses, a significance level of α = 0.05 was adopted. Descriptive analyses and chi-square adherence tests were performed by using the IBM SPSS Statistics software package, version 20.0 (IBM Corp., Armonk, NY, USA). The reproducibility analyses were performed by using R software, version 3.6.0 (The R Project for Statistical Computing, Vienna, Austria), with the irr, irrCAC, and rel packages.

## RESULTS

### Frequency of diagnostic MRI features

**Figure 1:**
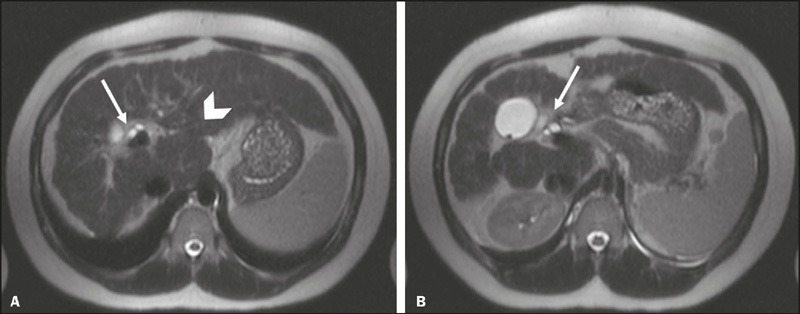
Frequent chronic hepatitis MRI findings in a 45-year-old woman with AIH. Axial T2-weighted MRI sequences showing surface nodularity, with an enlarged preportal space (arrow in A) and enlargement of the caudate lobe (arrowhead in A); and an expanded gallbladder fossa (arrow in B).

**Figure 2:**
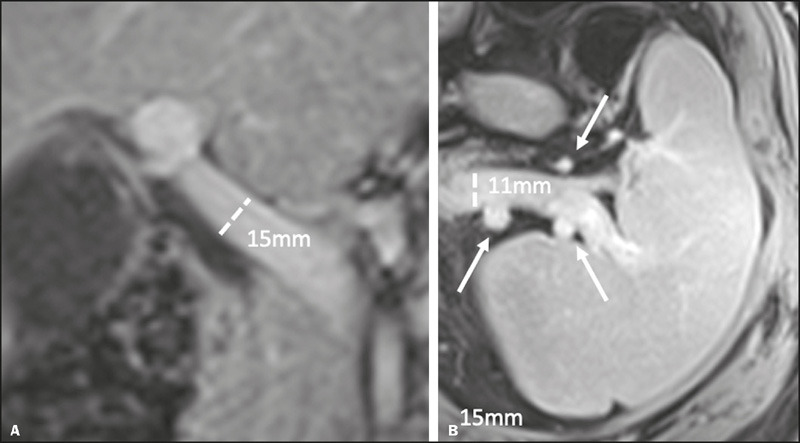
Portal hypertension secondary to CLD in a 50-year-old woman with AIH. Contrast-enhanced coronal and axial T1-weighted sequences (**A** and **B,** respectively), acquired in the portal phase. In **A,** an increase in the diameter of the portal vein is observed. In **B,** increased diameter of the splenic vein, collateral vessels in the splenic hilum (arrows), and splenomegaly (splenic index of 800) are shown.

Of the 20 patients evaluated, only three (15%) had no findings of cirrhosis. As depicted in Figure 1, we observed surface nodularity in 17 patients (85%), expanded gallbladder fossa in seven (35%), and enlarged preportal space in eight (40%). Findings of portal hypertension (Figure 2) included varices in seven patients (35%), ascites in seven (35%), and splenomegaly in 12 (60%). The splenic index ranged from 533 to 2548, with a mean of 734. Caudate lobe enlargement (Figure 1) was observed in 10 patients (50%).

**Figure 3:**
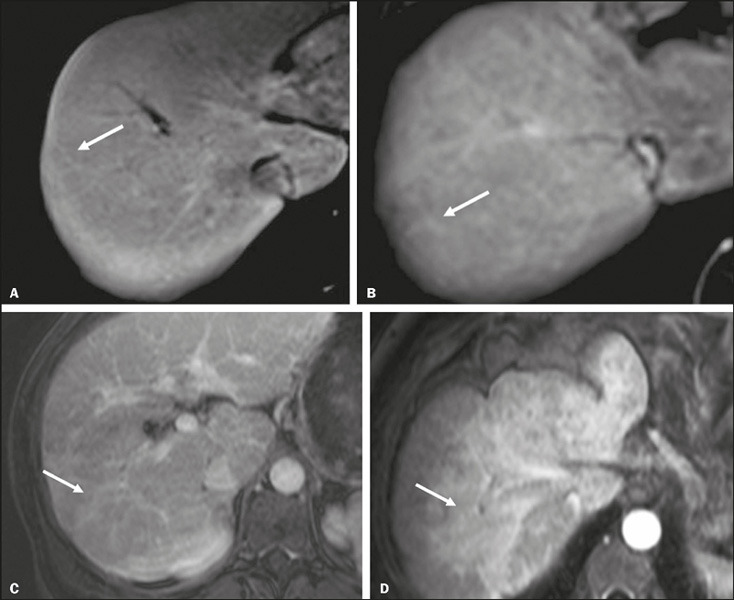
Grading of liver fibrosis by MRI in AIH. Fibrosis was characterized by lines with low signal intensity on an out-of-phase T1-weighted gradient-echo sequence (not included in the images), which show uptake of the paramagnetic contrast agent in the portal phase, exemplified in four patients with AIH (**A–D**). In **A,** discrete fibrosis with a reticular pattern (linear fibrotic tissue with diameter < 2 mm); in **B,** moderate fibrosis with a reticular pattern (fibrotic lines measuring between 2 mm and 5 mm); in **C,** severe fibrosis with a reticular pattern (thick fibrotic bands > 5 mm); and in **D,** an amorphous region of fibrosis > 2 cm, indicative of a confluent pattern of fibrosis.

Liver fibrosis (Figure 3) was observed in 16 (80%) of the patients, and a reticular pattern of fibrosis was observed in all of those cases: the pattern was exclusively reticular in 11 patients (55%) and was mixed (reticular and confluent) in five (25%). Among those 16 patients, the fibrosis was categorized as mild in four (25.0%), moderate in seven (43.8%), and severe in five (31.2%). In all of the patients with severe fibrosis, the pattern was mixed.

**Figure 4:**
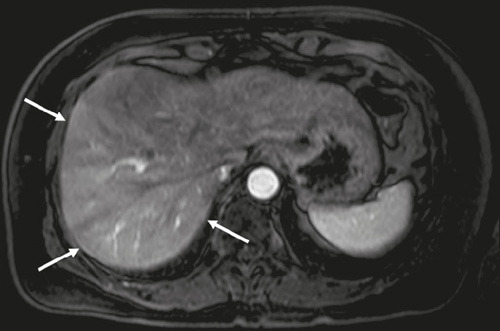
Heterogeneous enhancement of the liver parenchyma in AIH. Contrast-enhanced Tl-weighted MRI sequence, in the arterial phase, showing enhancement that is asymmetric (more intense in the right lobe), a finding that is reported in approximately one third of patients with AIH and can be attributed to hepatocellular inflammation/damage.

**Figure 5:**
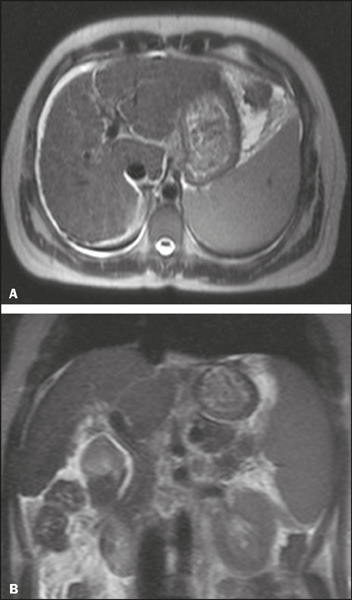
The most common volumetric change in AIH: diffuse atrophy. Axial and coronal T2-weighted MRI sequences (**A** and **B,** respectively) showing liver parenchymal volume below normal (estimated volume = 756 cm^3^).

As shown in Figure 4, heterogeneous liver enhancement was observed in 13 patients (65%). Liver volume was calculated for each patient, and global atrophy (Figure 5) was the most common finding. Liver volumes ranged from 685 mL to 1696 mL, with a mean of 1136 mL. Hepatic steatosis was observed in only one (5%) of the 20 patients. Hypervascular liver nodules were observed in two patients (10%), with diameters of 5 mm and 12 mm, respectively. None of our patients had hepatocellular carcinoma or venous thrombosis.

Intrahepatic biliary duct dilatation was observed in three (15%) of the 20 patients, involving the entire liver in two (10%), and lymphadenopathy was observed in eight patients (40%). Tables 1 and 2 summarize the descriptive analyses.

**Table 1:** MRI features in a sample of patients with AIH (N = 20).

Feature	n (%)
Fibrosis	
None	4 (20)
Reticular pattern only	11 (55)
Reticular and confluent patterns	5 (25)
Degree of fibrosis	
Mild (< 2 mm)	4 (25.0)
Moderate (2–5 mm)	7 (43.8)
Severe (> 5 mm)	5 (31.3)
Liver enhancement	
Homogeneous	7 (35)
Heterogeneous	13 (65)
Intrahepatic biliary duct dilatation	
None	17 (85)
Left lobe	0 (0)
Right lobe	1 (5)
Diffuse	2 (10)
Expanded gallbladder fossa	
No	13 (65)
Yes	7 (35)
Enlarged preportal space	
No	12 (60)
Yes	8 (40)
Caudate lobe enlargement	
No	10 (50)
Yes	10 (50)
Surface nodularity	
No	3 (15)
Yes	17 (85)
Hepatocellular carcinoma	
No	20 (100)
Yes	0 (0)
Hypervascular nodules*	
No	18 (94.7)
Yes	1 (5.3)
Hepatic steatosis	
No	19 (95)
Yes	1 (5)
Lymphadenopathy	
No	12 (60)
Yes	8 (40)
Ascites	
No	13 (65)
Yes	7 (35)
Portal vein thrombosis*	
No	19 (100)
Yes	0 (0)
Collateral vessels	
No	13 (65)
Yes	7 (35)
Splenomegaly	
No	8 (40)
Yes	12 (60)

* Dado não disponível de um paciente em razão de falha técnica na aquisição da sequência correspondente.

**Table 2:** Quantitative MRI features of AIH (N = 20).

Feature	Mean ± SD
Liver volume (cm^3^)	1,135.93 ± 295.56
Portal vein diameter (mm)	11.93 ± 1.515
Splenic vein diameter (mm)	8.38 ± 1.798
Splenic index	734.08 ± 292.874

### Interobserver agreement for MRI features

The interobserver agreement was excellent for surface nodularity (0.83), ascites (0.91), liver volume (0.95), intrahepatic biliary duct dilatation (0.84), and splenomegaly (0.81). Conversely, the interobserver agreement was just slight and fair for the degree of fibrosis and heterogeneous liver enhancement (0.12 and 0.25, respectively). It was also slight or fair for some CLD findings, such as expanded gallbladder fossa (0.14) and enlarged preportal space (0.36). Tables 3 and 4 summarize the interobserver agreement values.

**Table 3:** Interobserver agreement for qualitative MRI features.

Feature	AC1	95% CI
Portal vein thrombosis	1.00 (*p* < 0.001)	1.000-1.000
Hepatocellular carcinoma	1.00 (*p* < 0.001)	1.000-1.000
Hepatic steatosis	0.95 (*p* < 0.001)	0.832-1.000
Ascites	0.91 (*p* < 0.001)	0.707-1.000
Hypervascular nodules	0.88 (*p* < 0.001)	0.697-1.000
Intrahepatic biliary duct dilatation	0.84 (*p* < 0.001)	0.637-1.000
Surface nodularity	0.83 (*p* < 0.001)	0.597-1.000
Splenomegaly	0.81 *(p* < 0.001)	0.526-1.000
Collateral vessels	0.45 (*p* = 0.048)	0.003-0.896
Caudate lobe enlargement	0.41 (*p* = 0.070)	0.036-0.848
Enlarged preportal space	0.36 (*p* = 0.139)	0.128-0.848
Enhancement	0.25 (*p* = 0.324)	0.268-0.771
Expanded gallbladder fossa	0.14 (*p* = 0.600)	0.404-0.680
Degree of fibrosis	0.12 (*p* = 0.655)	0.455-0.693
Lymphadenopathy	0.04 (*p* = 0.876)	0.472-0.549	

**Table 4:** Interobserver agreement for quantitative MRI features.

Feature	Mean ± SD	ICC	95% CI
Liver volume (cm^3^)	1,135.93 ± 295.56	0.95 (*p* < 0.001)	0.883-0.980
Splenic index	734.08 ± 292.874	0.76 (*p* = 0.010)	0.147-0.935
Splenic vein diameter	8.38 ± 1.798	0.70 *(p* < 0.001)	0.370-0.868
Portal vein diameter	11.93 ± 1.515	0.43 (*p* = 0.051)	−0.071-0.750

## DISCUSSION

Whereas previous studies have addressed the frequency of morphological alterations in the liver across a diverse spectrum of etiologies, encompassing conditions ranging from alcohol-induced liver disease to viral hepatitis and nonalcoholic steatohepatitis^(6,7^, our focus in the present study was directed toward an entity that has received comparatively less attention: AIH. Subjective evaluation of the liver imaging with a focus on the etiology is paramount in the everyday practice of radiologists. Therefore, consolidated knowledge of the most common morphological changes in AIH is valuable when reading examinations of such a relatively uncommon disease. In addition, these basic studies hold the potential to unveil potential correlations between distinct morphological modifications and etiology, thereby contributing to the establishment of imaging hallmarks that are more precise for raising the suspicion of AIH. Seminal works in other conditions have found, for instance, that enlargement of the caudate lobe and the presence of the right posterior hepatic notch sign on MRI are seen more commonly in alcoholic cirrhosis than in virus-related cirrhosis^(6^, that morphometric changes of cirrhosis display different patterns according to their etiology, and that differences between etiologies decrease as cirrhosis progresses^(7^.

Our findings indicate that MRI features in AIH are related to CLD and are frequently observed, as well as that interobserver agreement in the MRI ana lysis of AIH patients is excellent for some major signs of CLD and its complications, especially those related to portal hypertension, such as surface nodularity, ascites, liver volume, and splenomegaly. Conversely, the level of interobserver agreement was lower for other frequent but less objective criteria, such as the degree of fibrosis, liver enhancement, expanded gallbladder fossa and enlarged preportal space. To our knowledge, there have been no previous studies reporting the reproducibility of the full spectrum of imaging findings in the context of AIH.

Classically, the predominant imaging feature of AIH is cirrhosis^(2,4,8,9^, and its presentation varies according to the chronicity (stage) of the disease^(9,24^. Even during treatment (corticosteroid therapy), liver fibrosis develops or progresses in at least a quarter of patients with AIH^(25^. In our sample, approximately 80% of the patients presented with liver fibrosis, a third of them at an advanced degree (concomitant severe reticular and confluent patterns). Regarding liver enhancement, heterogeneous enhancement was the most common pattern found. In CLD, heterogeneous liver enhancement on MRI has been associated with recent or concurrent hepatocellular damage^(26^.

We also found the interobserver agreement to be excellent for liver volume, ascites, and surface nodularity, whereas it was just slight and fair for the degree of fibrosis and for heterogeneous liver enhancement, respectively. Disagreements were also observed in the identification of an expanded gallbladder fossa and an enlarged preportal space, with only slight and fair agreement, respectively. In fact, the level of agreement between the two radiologists was lower for subjective criteria and higher for more objective, quantitative criteria, being excellent for liver volume and good for the splenic index. That suggests that subjective characteristics have a greater degree of difficulty and are more likely to result in disagreement. A recent study of patients with primary sclerosing cholangitis produced results similar to ours, showing that interobserver agreement was better for the identification of ascites, surface nodularity, hepatomegaly, and splenomegaly than for the characterization of heterogeneous enhancement of the parenchyma in the arterial phase^(27^. Our findings are also in accordance with those of another study, in which the authors assessed the performance of morphologic criteria for the diagnosis of cirrhosis^(28^, reporting that the imaging features for which the level of interobserver agreement was highest were ascites (κ = 0.85), splenomegaly (κ = 0.78), and surface nodularity (κ = 0.71). In that same study, the imaging features for which the level of interobserver was lowest were the caudate-to-right-lobe ratio (κ = 0.37), enlarged periportal space (κ = 0.31), and expanded gallbladder fossa (κ = 0.23).

Another point to emphasize is the challenge posed by the characterization of portal hypertension on MRI, as in our study. There is as yet no consensus regarding the cutoff values for portal and splenic vein diameters for cross-sectional ultrasound imaging, with conflicting and still emerging evidence in the literature. For instance, Stamm et al.^(29^ found that the normal main portal vein diameter, as measured on CT, is larger than the widely referenced upper limit of 13 mm. Recently, Huang et al. published updated reference values for four-dimensional flow MRI of the portal venous system, with mean portal vein diameters ranging from 15.8 ± 2.4 mm to 16.4 ± 2.3 mm, depending on the portal vein segment^(30^. A recent systematic review and meta-analysis of portal vein mor-phometry in pediatric and adult populations showed that the portal vein diameter was significantly larger when measured by CT than when measured by other imaging modalities^(31^: 13.28 mm (95% CI: 11.71–14.84) versus 10.50 mm (95% CI: 9.35–11.66) for MRI and 9.81 mm (95% CI: 9.47–10.16) for ultrasound. That proximity between the ultrasound and MRI values might validate our approach and that of other authors employing such cutoff values, although this is still an open question.

Grading fibrosis by imaging methods in CLD has been the focus of recent research^(32^. Although liver biopsy is considered the gold standard, the high cost, limited availability, and invasive nature of the procedure make it impractical in some cases. Although attempting to grade fibrosis by using conventional imaging methods is an important step, it remains challenging. In fact, the conventional MRI fibrosis grading used in the present study, albeit relevant, seems to be very subjective and difficult to apply in practice, with considerable disagreement between readers (with only slight interobserver agreement). Advanced quantitative imaging methods (such as ultrasound and magnetic resonance elastography) provide more objective evaluations, can improve diagnostic accuracy, and should be considered for the grading of fibrosis in all forms of CLD, including AIH, in clinical practice^(33,34,35^. In this context, we highlight a recent study in which quantitative MRI parameters (T1 mapping and extracellular volume fraction) alone showed excellent performance in diagnosing significant fibrosis (≥ F2) in patients with AIH^(35^. Further studies should be conducted to explore these features in evaluating different stages of the disease. Another possible use for imaging in AIH is in the treatment followup, as a means of avoiding the need for serial biopsies for monitoring treatment response; recent studies have highlighted the potential of advanced MRI sequences for that task. For instance, in a prospective study of 62 patients who underwent an MRI scan at recruitment and after 12–18 months, Arndtz et al.^(36^ found an association between T1 mapping values and recurrence after remission. Those authors also found that T1 mapping values at baseline were a significant predictor of recurrence after biochemical remission.

Our study has some limitations. The number of patients was small, which makes identifying statistically significant trends and correlations difficult. However, AIH is a rare disease that is not frequently evaluated by imaging examinations in medical practice. Nevertheless, our sample size was at least similar to or larger than those of previous studies^(8,9^. In addition, we assessed reproducibility by considering the analyses of only two readers, although this approach has been taken in studies of other conditions^(27,37^. Although interobserver agreement was reported for a few variables in a previous study of AIH patients^(8^, this is, to our knowledge, the first study to assess reproducibility across the full spectrum of imaging findings in AIH. Studies considering the analyses of a larger number of readers with different degrees of experience could be helpful. Furthermore, we did not correlate the histopathological degree of fibrosis with the fibrosis grading proposed in our imaging criteria evaluation, because our study encompassed a considerable period of time and because the fibrosis status of a given patient at the time of MRI examination cannot necessarily be correlated with the fibrosis at the time of biopsy (the evolution over time and the effects of treatment could affect the precision of such analysis). Nevertheless, to our knowledge, the present study involved the largest sample of patients with biopsy-proven AIH evaluated to date. There is a need for additional studies to determine whether the extent of fibrosis in AIH, as measured by MRI, increases or decreases in response to corticosteroid therapy. Moreover, we did not employ the most recent MRI techniques for quantifying fibrosis, such as magnetic resonance elastography and techniques involving the use of hepatobiliary contrast agents (e.g., T1 mapping), because such techniques were not widely available during the entire period of data collection. However, we are currently conducting a prospective study addressing that topic. Finally, the evaluation of other relevant prognostic criteria, such as signs of portal hypertension, was also somewhat limited in our study, given the lack of any clinical or histological correlation.

In conclusion, our study demonstrates that MRI can correctly identify classic morphologic signs of cirrhosis and portal hypertension, which are the most common MRI findings in AIH, constituting a critical step in the assessment and risk stratification of these patients. However, the interobserver agreement for individual signs ranged from fair to excellent, the lowest agreement being related to subjective features. These aspects underscore the importance of using imaging-based methods that are more objective and more advanced, especially for grading inflammation and fibrosis, in AIH. Although not the main focus of this work, imaging may also contribute to defining the diagnosis in the early stages of the disease, principally in differentiating between AIH and other liver diseases, given that, in the advanced stages (after cirrhosis has become established), the imaging findings of AIH are not expected to present a substantial difference from those of other etiologies.
